# Supplementary material for: mutscan—a flexible R package for efficient end-to-end analysis of multiplexed assays of variant effect data
Source: Genome Biol. 2023 Jun 1;24:132. doi: 10.1186/s13059-023-02967-0 (PMC10236832; doi:10.1186/s13059-023-02967-0)
Supplement: Supplementary file 3 — Additional file 3. Review history. [file 13059_2023_2967_MOESM3_ESM.docx]

Review History

**First round of review**

**Reviewer 1**

**Were you able to assess all statistics in the manuscript, including the appropriateness of statistical tests used?** Yes, and I have assessed the statistics in my report.

**Were you able to directly test the software?** No

**Comments to author:**

STRENGTHS

1. (Significance) This paper introduces a new computational pipeline to process large-scale MAVE datasets, with improved computational efficiency and less memory usage, and a more flexible interface to process Fastq files that is compatible with a wide range of MAVE libraries.

2. (Clarity) The paper is overall well written and includes the table of high informativeness, which really spells out exactly the contributions that are made compared to the previous two methods.

WEAKNESS

1. (Results) The paper shows that the results of mutscan are highly concordant with those of other methods, which is encouraging. However, it would be helpful to further demonstrate that these results are meaningful and consistent with established biological mechanisms. One way to do this could be to compare the heatmap in Fig. 2F with metrics of sequence conservation and covariation, or to construct a log representation based on the output of mutscan and compare it to the FOS/JUN binding motif. These types of analyses would provide stronger evidence for the validity of the mutscan results.

2. (Results) Although log-fold changes often correlate with fitness scores (Fig. 6), the relationship between this intrinsic property and the measured fold-changes can be complicated and nonlinear [1,2]. It's not always appropriate to directly use the log-fold changes "as fitness scores for downstream interpretation" as suggested in the method. This section would benefit from further explanation or demonstration on how to effectively use the output from mutscan to identify significant genetic variants.

(1) Tareen, A., Kooshkbaghi, M., Posfai, A. et al. MAVE-NN: learning genotype-phenotype maps from multiplex assays of variant effect. Genome Biol 23, 98 (2022).

(2) Kinney JB, McCandlish DM. Massively Parallel Assays and Quantitative Sequence-Function Relationships. Annu Rev Genomics Hum Genet 20, 99 (2019).

MINOR POINTS

1. MAVE is an umbrella term that encompasses methods such as Deep Mutational Scanning (DMS) and massively parallel reporter assays (MPRAs). However, the datasets presented in the paper primarily seem to be from DMS experiments.

2. Some of the figure legends are scarce and difficult to understand directly.

3. It is interesting that one of the datasets in Fig. 6 shows lower correlation between methods. It would be useful to explore a bit more whether the underlying framework can provide any insight into this discrepancy.

**Reviewer 2**

**Were you able to assess all statistics in the manuscript, including the appropriateness of statistical tests used?** Yes, and I have assessed the statistics in my report.

**Were you able to directly test the software?** Yes

**Comments to author:**

Review of mutscan - flexible R package for efficient end-to-end analysis of MAVE

This package does what it was designed to do and it does it very fast. Its lack of dependencies did allow our tester to get it running in a reasonable amount of time without much frustration (bioconductor gave us issues, but that is our problem). The digestfastq() function has many useful parameters that allow users to eliminate or simplify the pre-processing steps usually associated with NGS datasets. We also really liked the ability to concatenate multiple runs, it is quite an improvement over other MAVE software packages.

This package is good at processing data for one type of MAVE experiment - a defined-plasmid-based library in a simple selection assay; however it is not as flexible as one might hope. The MAVE field has moved toward more complex ways of making perturbations, such as genome editing by both HDR and base editing, meaning that the ability to properly handle indels and using gapped alignment algorithms and not string matching is becoming increasingly important. And assays are getting more complicated - some require comparison across time points, multiple FACS bins and even single cell transcriptomes. Mutscan is not designed to handle generating a score for these cases, but, one could use mutscan count and call the variants for such experiments, then use other R functions to generate a score since the dataframe is within the R environment which is nice.

Overall the summary table contains a lot of useful information. The counts of mutant codons as well as the amino sequence and mutation types is useful, but the mutant naming syntax and variant calls are difficult to parse. Additionally, expressing the data as HGVS strings to match to human mutational data would be quite useful as well as linking to genomic coordinates.

Figuring out which parameters to choose is challenging. The example datasets are great but the examples of each function could be improved.

Overall, I think this R package will be useful to the field. It is an improvement upon DimSum and Enrich 2, which I know that many are still using.

addition question:

Fig 6. What is so different about MutScan and DimSum in Diss_FOS_JUN and the Bolognesi data sets? The explanation in the text is not sufficient.

**Authors Response**

**Point-by-point responses to the reviewers’ comments:**

We would like to thank the reviewers for their positive assessment of mutscan and their constructive comments. Below we provide point-by-point responses to each of the points raised by the reviewers, and all changes to the manuscript main text are highlighted in yellow.

Reviewer #1: STRENGTHS

1. (Significance) This paper introduces a new computational pipeline to process large-scale MAVE datasets, with improved computational efficiency and less memory usage, and a more flexible interface to process Fastq files that is compatible with a wide range of MAVE libraries.
2. (Clarity) The paper is overall well written and includes the table of high informativeness, which really spells out exactly the contributions that are made compared to the previous two methods.

WEAKNESS

1. (Results) The paper shows that the results of mutscan are highly concordant with those of other methods, which is encouraging. However, it would be helpful to further demonstrate that these results are meaningful and consistent with established biological mechanisms. One way to do this could be to compare the heatmap in Fig. 2F with metrics of sequence conservation and covariation, or to construct a log representation based on the output of mutscan and compare it to the FOS/JUN binding motif. These types of analyses would provide stronger evidence for the validity of the mutscan results.

We thank the reviewer for this comment. We agree that showing how these heatmaps can help interpreting the mechanisms of mutation effects would be useful for the reader. We thus added a paragraph with an extended interpretation of the results in Fig. 2F:

“These heatmaps serve as the basis for interpreting the mechanisms by which mutations impact the molecular activity studied. For instance, ...”

We think that making additional interpretations that go beyond the ones made in the original paper (Diss and Lehner, eLife, 2018) on the basis of an equivalent heatmap is beyond the scope of this manuscript.

2. (Results) Although log-fold changes often correlate with fitness scores (Fig. 6), the relationship between this intrinsic property and the measured fold-changes can be complicated and nonlinear [1,2]. It's not always appropriate to directly use the log-fold changes "as fitness scores for downstream interpretation" as suggested in the method. This section would benefit from further explanation or demonstration on how to effectively use the output from mutscan to identify significant genetic variants.

We thank the reviewer for this comment, which we believe reveals a confusion due to the use of the word “fitness” in our manuscript. We did not intend to suggest that log-fold changes should be interpreted directly as (molecular) fitness scores. What we meant by "as fitness scores for downstream interpretation" was that the log-fold changes are often also referred to as “fitness scores”, for example in the paper describing DiMSum (Faure et al., Genome Biology, 2020). As the reviewer rightfully notes, these fitness scores or log-fold changes do not necessarily correlate linearly to molecular fitness or molecular activity such as for instance binding free energy. To avoid the confusion, we replaced all instances of “fitness scores” in the manuscript by “log-fold changes” or “enrichment scores”. We also reworded the sentence in the Methods section to “The log-fold changes returned by these models can be used as a proxy for molecular activity for downstream interpretation”.

(1) Tareen, A., Kooshkbaghi, M., Posfai, A. et al. MAVE-NN: learning genotype-phenotype maps from multiplex assays of variant effect. Genome Biol 23, 98 (2022).
(2) Kinney JB, McCandlish DM. Massively Parallel Assays and Quantitative Sequence-Function Relationships. Annu Rev Genomics Hum Genet 20, 99 (2019).

MINOR POINTS

1. MAVE is an umbrella term that encompasses methods such as Deep Mutational Scanning (DMS) and massively parallel reporter assays (MPRAs). However, the datasets presented in the paper primarily seem to be from DMS experiments.

The reviewer is correct that the selected examples are all from DMS experiments. We are however not aware of any fundamental differences between DMS experiments and more general MAVE experiments that would preclude their analysis using *mutscan*. For that reason, we refer to the umbrella term in our manuscript.

2. Some of the figure legends are scarce and difficult to understand directly. Thank you for the comment. We agree and have made the figure legends more extensive.
3. It is interesting that one of the datasets in Fig. 6 shows lower correlation between methods. It would be useful to explore a bit more whether the underlying framework can provide any insight into this discrepancy.

The reviewer is correct that for some datasets, the correlations between the different tools turns out lower. This is due to less stringent filtering in these datasets, which retain many variants with more mutations and lower counts in the analysis. The observed deviations are dominated by these low-abundance variants, which is also demonstrated in Figures S11-S14, where the correlations are stratified by the number of observed mutations. To clarify this effect, we added the following sentences to the legend of Figure 6:

“...The lower correlations seen in the Bolognesi_TDP43_290_331 dataset and to some extent the Diss_FOS_JUN dataset reflect the less stringent variant filtering criteria used in these datasets (up to 3 mutated bases for Bolognesi_TDP43_290_331 and up to two mutated codons for Diss_FOS_JUN, see Methods). Considering only variants with fewer recorded mutations leads to higher correlations, more comparable e.g. to the Diss_FOS dataset where only up to two mutated bases were allowed (Additional file 2:Figures S11-S14).”

Reviewer #2: Review of mutscan - flexible R package for efficient end-to-end analysis of MAVE

This package does what it was designed to do and it does it very fast. Its lack of dependencies did allow our tester to get it running in a reasonable amount of time without much frustration (bioconductor gave us issues, but that is our problem). The digestfastq() function has many useful parameters that allow users to eliminate or simplify the pre-processing steps usually associated with NGS datasets. We also really liked the ability to concatenate multiple runs, it is quite an improvement over other MAVE software packages.

This package is good at processing data for one type of MAVE experiment - a defined-plasmid-based library in a simple selection assay; however it is not as flexible as one might hope. The MAVE field has moved toward more complex ways of making perturbations, such as genome editing by both HDR and base editing, meaning that the ability to properly handle indels and using gapped alignment algorithms and not string matching is becoming increasingly important.

We agree with the reviewer that currently, *mutscan* has not been designed to handle insertions or deletions in an efficient way. In fact, adding support for indels to *mutscan* is high on our list of planned additions to the tool, but beyond the scope of the current manuscript, as it will take substantial refactoring of the current code. We believe that *mutscan* is still a useful contribution to the field in its current form, given that the vast majority of public MAVE experiments are based on substitution variants.

And assays are getting more complicated - some require comparison across time points, multiple FACS bins and even single cell transcriptomes. Mutscan is not designed to handle generating a score for these cases, but, one could use mutscan count and call the variants for such experiments, then use other R functions to generate a score since the dataframe is within the R environment which is nice.

While the calculateFitnessScore() function is intended for a very specific experimental setup (paired input-output samples and a growth-rate based readout), *mutscan* also contains a more general statistical testing framework, provided by the calculateRelativeFC() function, which builds on the *edgeR* and *limma* packages and therefore can accommodate any fixed effect experimental design, including designs with multiple time points, FACS bins and additional covariates. For example, in a time course experiment, calculateRelativeFC() can be used to compare each individual time point to any other time point, either individually or in combination. Or similarly, assuming a constant rate of change over time, calculateRelativeFC() can be used to combine all time points and estimate that rate.

Overall the summary table contains a lot of useful information. The counts of mutant codons as well as the amino sequence and mutation types is useful, but the mutant naming syntax and variant calls are difficult to parse. Additionally, expressing the data as HGVS strings to match to human mutational data would be quite useful as well as linking to genomic coordinates.

Linking the observed variants to genomic coordinates would be challenging for *mutscan*, as it would require providing additional information on the provided wildtype sequences and their mapping to genomic coordinates.

We do agree with the reviewer that supporting established variant naming conventions would be a useful feature. We have therefore extended *mutscan’s* digestFastq() function to include additional variant names in its output. The output now contains HGVS-conforming names for both the nucleotide and amino acid version of each variant. If the user provided appropriately named wildtype sequences (following HGVS nomenclature), these names will correspond to valid HGVS identifiers. We hope that this will make the matching to human reference mutation databases easier and our tool more useful.

Figuring out which parameters to choose is challenging. The example datasets are great but the examples of each function could be improved.

Thank you for this comment. We have added examples to the documentation page for each function, exemplifying different use cases (see <https://fmicompbio.github.io/mutscan/reference/index.html>). We refer to the vignette (<https://fmicompbio.github.io/mutscan/articles/mutscan.html>) for longer-form, worked out examples of the full analysis pipeline for different types of experiments.

Overall, I think this R package will be useful to the field. It is an improvement upon DimSum and Enrich 2, which I know that many are still using.

addition question:

Fig 6. What is so different about MutScan and DimSum in Diss_FOS_JUN and the Bolognesi data sets? The explanation in the text is not sufficient.

We agree with the reviewer on this point, which has also been brought up by reviewer 1 (minor point 3). We would like to refer to our response to that point above.

**Second round of review**

**Reviewer 1**

We thank the authors for their detailed response and effort on improving their manuscript and method. We think the authors did a good job with the revision improving the manuscript and adding features to their package. While it is still generally desirable to offer additional lines of evidence beyond the mere correlation with other MAVE tools, we acknowledge the authors' efforts in explaining their present findings, and agree that the method demonstrates efficiency and flexibility compared to other MAVE software tools. With the additional clarification from authors, we believe there is a strong case for this work and will be updating our score accordingly. We congratulate the authors on a job well done addressing our and the other reviewers' comments.

**Reviewer 2**

My concerns have been addressed.
